# Effects of Deletion of Mutant Huntingtin in Steroidogenic Factor 1 Neurons on the Psychiatric and Metabolic Phenotype in the BACHD Mouse Model of Huntington Disease

**DOI:** 10.1371/journal.pone.0107691

**Published:** 2014-10-01

**Authors:** Barbara Baldo, Rachel Y. Cheong, Åsa Petersén

**Affiliations:** Translational Neuroendocrine Research Unit, Department of Experimental Medical Science, Lund University, Lund, Sweden; Boston University School of Medicine, United States of America

## Abstract

Psychiatric and metabolic features appear several years before motor disturbances in the neurodegenerative Huntington’s disease (HD), caused by an expanded CAG repeat in the *huntingtin* (*HTT*) gene. Although the mechanisms leading to these aspects are unknown, dysfunction in the hypothalamus, a brain region controlling emotion and metabolism, has been suggested. A direct link between the expression of the disease causing protein, huntingtin (HTT), in the hypothalamus and the development of metabolic and psychiatric-like features have been shown in the BACHD mouse model of HD. However, precisely which circuitry in the hypothalamus is critical for these features is not known. We hypothesized that expression of mutant HTT in the ventromedial hypothalamus, an area involved in the regulation of metabolism and emotion would be important for the development of these non-motor aspects. Therefore, we inactivated mutant HTT in a specific neuronal population of the ventromedial hypothalamus expressing the transcription factor steroidogenic factor 1 (SF1) in the BACHD mouse using cross-breeding based on a Cre-loxP system. Effects on anxiety-like behavior were assessed using the elevated plus maze and novelty-induced suppressed feeding test. Depressive-like behavior was assessed using the Porsolt forced swim test. Effects on the metabolic phenotype were analyzed using measurements of body weight and body fat, as well as serum insulin and leptin levels. Interestingly, the inactivation of mutant HTT in SF1-expressing neurons exerted a partial positive effect on the depressive-like behavior in female BACHD mice at 4 months of age. In this cohort of mice, no anxiety-like behavior was detected. The deletion of mutant HTT in SF1 neurons did not have any effect on the development of metabolic features in BACHD mice. Taken together, our results indicate that mutant HTT regulates metabolic networks by affecting hypothalamic circuitries that do not involve the SF1 neurons of the ventromedial hypothalamus.

## Introduction

Huntington’s disease (HD) is a devastating neurodegenerative disorder caused by an expanded CAG repeat in the *huntingtin* (*HTT*) gene [1]. It is clinically diagnosed when an individual presents with typical involuntary movements in combination with a positive gene test. The disease then progresses over 15–25 years, inevitably leading to death. It is now well recognized that individuals with the mutant HTT gene often manifest psychiatric and cognitive disturbances many years before the motor onset of the disease [2], [3]. Other non-motor features are also common including metabolic dysfunction, changes in the circadian rhythm as well as sleep disturbances [4]. The motor symptoms and the cognitive changes have been associated with the pathology in the basal ganglia as well in the cerebral cortex [5], [6], but less is known about the neural substrate of other non-motor aspects of the disease.

The hypothalamus is a region in the brain involved in the regulation of important functions such as emotion, metabolism and circadian rhythm [7]. Neuropathological analyses have revealed effects on different neuropeptide populations regulating emotion and metabolism in human postmortem tissue from HD patients [8]–[11]. Hypothalamic changes have indeed been shown in clinical HD prior to motor dysfunction using magnetic resonance imaging (MRI) and positron emission tomography (PET) [12], [13], suggesting that this region may be affected early in the HD process. Interestingly, recent studies in experimental models of HD have established a direct link between the expression of mutant HTT in the hypothalamus and the development of non-motor signs [14], [15]. Expression of mutant HTT by the use of stereotactic injections of recombinant adeno-associated viral (rAAV) vectors in the hypothalamus of wild-type (WT) mice led to increased body weight as well as insulin and leptin resistance. Another experiment used the BACHD mouse model of HD expresses, which human full-length mutant HTT with a floxed exon1 with 97 CAG, allowing selective deletion of mutant HTT in the presence of Cre-recombinase (Cre; [16]). The mouse model displays both depressive and anxiety-like behaviors as well as a robust metabolic phenotype with leptin and insulin resistance at an early age [14], [17]. Interestingly, inactivation of mutant HTT in the hypothalamus of the BACHD mouse using rAAV-Cre prevented the development of both the metabolic phenotype as well as the depressive-like behavior [14], [15]. These studies were conducted by either expressing or inactivating mutant HTT in around 30% of the neurons in the hypothalamus [14], [15]. Additional experiments carried out by inactivating mutant HTT only in leptin receptor-expressing neurons in the hypothalamus of BACHD mice did not have any effect on the phenotype [18]. Hence, the critical neuronal circuitry in the hypothalamus responsible for the development of the non-motor features remains to be determined.

The ventromedial hypothalamus (VMH) plays a crucial role in the control of metabolism, energy expenditure and anxiety-like behavior [19], [20]. The neurons expressing steroidogenic factor 1 (SF1) represent a large part of the neurons in the VMH [21]. SF1 is a transcription factor belonging to the subfamily of transcription factors known as nuclear receptor subfamily 5 and has a fundamental role for VMH development [22]–[26]. Most importantly, SF1-expressing neurons contribute in the regulation of energy homeostasis and body weight mainly through the leptin circuitry [27]–[30]. Along with their key role in the regulation of metabolism, SF1-expressing neurons have been proposed to control the development of psychiatric-like behavior since SF1 controls the transcription of cannabinoid receptor 1 and SF1 knock-out mice present anxiety-like features [21], [23], [29]. Therefore, we hypothesized that expression of mutant HTT in the SF1-expressing neuronal population of the VMH would be critical for the development of the metabolic and emotional dysfunction in BACHD mice. In order to answer this question, we crossed BACHD mice with animals expressing Cre under the SF1 promoter and performed a battery of behavioral and metabolic analysis at 4 months of age. Our results suggest that deletion of mutant HTT in the VMH and specifically in the SF1-expressing neurons is not sufficient for preventing the onset of metabolic and psychiatric-like signs in BACHD mice.

## Materials and Methods

### Ethics statement

All the experimental procedures were approved by the Regional Ethical Committee in Lund, Sweden (Permit number: M360-12).

### Animals

BACHD mice, expressing human full-length HTT with 97 polyQ (FVB/N strain) and Tg(Nr5a1-Cre)7Lowl/J mice expressing Cre under the SF1 promoter (SF1; mixed FVB/N and C57BL6/J background) were obtained from Jacksons Laboratories (Bar Harbor, Maine, USA) [27]. Heterozygous SF1 males were crossed with FVB/N females obtained from Charles Rivers Laboratories (Sulzfeld, Germany). SF1 mice have no overt phenotype. SF1 females from the F1 generation were further crossed with BACHD males in order to obtain the generation used for all the experiments in this study (See Figure S1). The genotype of the offspring was determined from tail samples using PCR primers for Cre (5′–3′): forward GCGGTCTGGCAGTAAAAACTATC and reverse GTGAAACAGCATTGCTGTCACTT or for the BACHD transgene (5′–3′): forward CCGCTCAGGTTCTGCTTTTA and reverse AGGTCGGTGCAGAGGCTCCTC. The mice obtained from the F1 generation of the crossing BACHD-SF1 was tested for multiple behavioral and metabolic readouts (see paragraphs below) at 4 months of age. It is known that BACHD mice manifest psychiatric-like behaviors as well as metabolic changes as early as at 2 months of age and this phenotype is maintained until at least 12 months of age [14], [15], [31], [32]. The number of animals for each genotype and sex was between 11 and 28 in all experimental groups. The animals were culled at 4 months of age by decapitation for the collection of fresh frozen tissue or an overdose of anesthesia with sodium-pentobarbital (Apoteksbolaget) for perfusion with paraformaldehyde (PFA). All mice were housed in groups at a 12 h light/dark cycle with ad libitum access to normal chow diet.

To verify the selective expression and activity of Cre in the VMH, SF1 animals were crossed with B6.129X1-Gt(ROSA)26Sortm1(EYFP)Cos/J (ROSA-EYFP) and yellow fluorescent protein (EYFP) expression was evaluated by immunofluorescence (see paragraph below). ROSA-EYFP animals present a loxP-flanked STOP codon inserted before the EYFP gene, in the Gt(ROSA)26Sor locus, therefore expressing EYFP only in presence of active Cre-recombinase (C57BL/6 strain; Jackson laboratories).

### PCR for Cre-recombinase excision validation

In order to verify the successful deletion of mutant HTT exon 1 in BACHD-SF1 animals, we performed PCR analysis from genomic DNA extracted from selected brain regions. 300 µm thick slices of fresh frozen tissue from brains of animals belonging to each of the four genotypes were cut using a cryostat. Selected brain regions were rapidly collected using a puncher of 2 mm diameter (Miltex). Genomic DNA was extracted from the tissue with the DNeasy Blood and Tissue kit (Qiagen) according to manufacturer’s instructions. 1 µl of DNA was the used for the PCR reaction, which was performed using a PX2 Thermal Cycler (Thermo Electron Corporation). The PCR products were run on a 1% agarose gel for 40 min, with SYBR Safe DNA gel stain (Invitrogen). The primers were designed to detect the loxP flanked HTT exon 1 of the BACHD mice. Forward primer 5′-ATTCATTGCCCCGGTGCTGA-3′ and reverse primer 5′-AGCCCTCTTCCCTCTCAGACTAGAAGAGG-3′.

### Metabolic Analyses

Body weight of the animals and content of body fat were measured at 4 months of age. The body fat content was assessed under isoflurane anesthesia using a whole body dual energy x-ray absorptiometry (DEXA) scanner (Lunar PIXImus2, Lunar Corporation, Madison, WI, USA). Percentage of body fat was then calculated with the PIXImus2 2.10 software (Lunar Corporation, Madison, WI, USA).

Whole blood was collected from the right ventricle during deep anesthesia using an 18G syringe needle and kept at room temperature for 30 min allowing the blood to clot. The blood was then spun for 15 min at 2000×g and the serum collected and stored at −80°C until further analysis. Serum levels of leptin and insulin were measured using ELISA kits (CrystalChem Inc.) according to manufacturer’s instructions. Samples were diluted according to need and measured in duplicates.

### Behavioral Analyses

Animals belonging to the four genotypes were assessed for general activity as well as anxiety- and depressive- like behavior at 4 months of age. All behavioral tests were performed during the light phase of the circadian rhythm according to procedures described previously. All arenas were wiped with ethanol after each trial.

Total activity was assessed with the Open field test (OF) using the PAS-Open field system (San Diego Instruments) as previously described [15]. The animals were placed in the center of the activity box (40.6×40.6×38.1 cm) and their movements across the arena were recorded for 1 hour and scored as infrared beam crossings (16×16 beams).

Elevated Plus Maze (EPM) and Novelty-induced suppressed feeding test (NSFT) were used to assess anxiety-like behaviors [15], [33], [34]. EPM was performed in using the Ethovision 3.1 Software system (Noldus Information Technology). The EPM consisted of four 30 cm long and 6 cm wide arms (two open and two closed) and it was elevated 50 cm above its base. The closed arms were surrounded by 30 cm high walls. Each mouse was placed in the center of the maze and its movements recorded for 5 minutes. The Ethovision software calculated the percentage of time spent on the open or closed arms over this time interval.

NSFT was performed using an arena constituted by a plastic box (90×90 cm), with about 40 cm high walls and containing a light (approx. 800 lux) directed to the center of the arena, where the food pellet was located. Prior to the trial, the animals were food-deprived for 12 hours but they had ad libitum access to water. The body weight was measured pre- and post-food deprivation. Individual animals were placed in one corner of the arena and the latency to approach and eat the food was measured within a 5 min time frame. When the animal would start feeding it would be removed from the arena and placed back into the home cage. There they would be allowed to feed for 5 minutes from a single food pellet with known weight. The latency to eat in the home cage was measured and the food pellet weighed, to determine the food consumption in 5 min.

The Porsolt Forced Swim Test (FST) was used to assess depressive-like behavior. Each mouse was placed in a 17 cm wide and 18 cm high glass cylinder filled with 10–12 cm of water at 25°C. The mice were filmed for 6 min with a digital video camera and then dried with a towel. The total time spent immobile during the last 4 min of the trial was calculated manually. Immobility was defined by an absence of swimming movements (movements of two or more paws). Slow movements of a single paw aimed to keep the head above the water surface were included in the time of immobility, as described by Porsolt [35]. The mice were scored blinded by the same rater.

### Immunofluorescence and confocal imaging

In order to verify the presence of functional Cre activity, we crossed ROSA-EYFP mice with SF1 and visualized EYFP expression in the VMH. The cull of the mice was performed by transcardial perfusion with 4% PFA after deep anesthesia with sodium-pentobarbital (Apoteksbolaget). After 24 h post-fixation in 4% PFA, the brains were transferred to a 25% sucrose solution for cryopreservation and cut in 30 µm thick slices on a freezing microtome. Free floating slices were then processed with immunofluorescence for EYFP expression. After 1 h blocking with 5% normal donkey serum (NDS) in 0.25% Triton-X in KPBS (KPBS-T), the sections were incubated overnight in primary antibody chicken anti-GFP (*Abcam*, 1∶100000) in 5% NDS in KPBS-T at room temperature. The sections were washed 3 times in KPBS-T and then incubated with DyLight 488-conjugated AffiniPure donkey anti-chicken (*Jackson ImmunoResearch Laboratories, Inc.*, 1∶200) for 2 h at room temperature. After a final washing in KPBS the sections were mounted on gelatin-coated glass slides (Thermo Scientific) and cover-slipped with PVA-DABCO. The glasses were kept at 4°C in the dark till the acquisition of the images. GFP expression was visualized using a Nikon inverted confocal microscope with a 488 nm laser (*Coherent Sapphire)*. Z-stacks images were acquired using a 4X (NA 0.13) objective and NIS Elements (Nikon) software.

### Statistical Analyses

The data are presented as mean+SEM. Statistical analyses were performed using Prism 6. Statistical difference was considered at p<0.05. Normal distribution of the data was first verified using the Kolmogorov–Smirnov test. When normally distributed, the data were analyzed using one-way analysis of variance (ANOVA) followed by Tukey post hoc test. When not normally distributed, the data were analyzed using Kruskal–Wallis one-way analysis of variance followed by Dunn’s post hoc test. Power analysis was performed using the program PS: Power and Sample Size Calculation version 3.0, 2009 (http://biostat.mc.vanderbilt.edu/wiki/Main/PowerSampleSize). Further details on statistical analyses are available in Statistical Results S1.

## Results

### No effect of inactivation of mutant HTT in SF1-expressing neurons on the metabolic phenotype of BACHD mice

**Figure 1:**
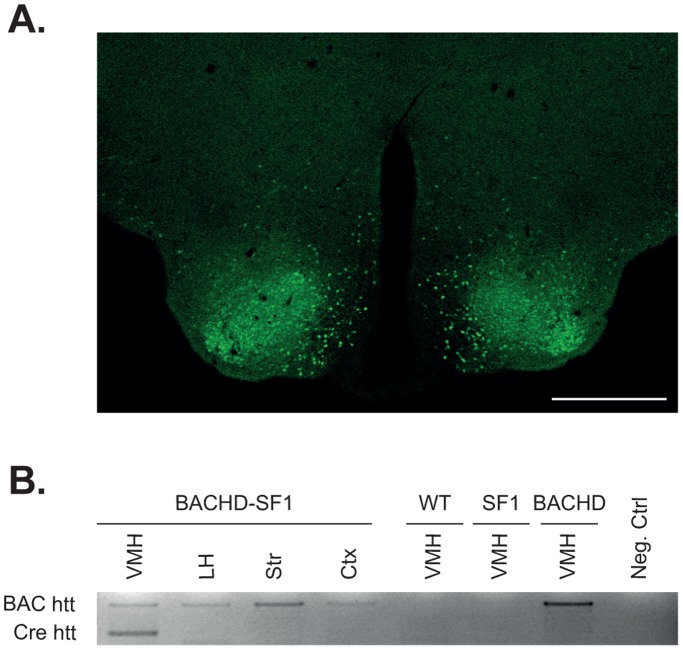
Validation of Cre-excision in SF1-expressing neurons in the VMH. A. SF1 mice were crossed with ROSA-EYFP mice and the expression in SF1 neurons was validated using GFP immunofluorescence. GFP immunoreactive cells were primarily clustered in the VMH. Scale bar represents 500 µm. B. Gel showing Cre recombination as validated by PCR of several brain regions from BACHD-SF1, BACHD, SF1 and WT mice. The presence of the unrecombined mutant HTT allele (1050 bp) was detected in all the BACHD-SF1 and BACHD samples while the recombined mutant HTT allele (600 bp) was only detected in VMH brain samples from the BACHD-SF1 mice.

To examine the role of SF1-expressing neurons in the development of metabolic and psychiatric impairments in HD, female SF1 mice were crossed with male BACHD mice to generate mice with a conditional deletion of mutant HTT from SF1-expressing neurons (Figure S1). We first verified the selective expression of Cre in SF1 neurons by crossing SF1 mice with ROSA-EYFP mice and assessed the degree of EYFP expression using immunofluorescence. We confirmed that EYFP-expressing neurons were localized in the VMH (Figure 1A). In addition, the region specificity of Cre recombination resulting in the excision of exon 1 of mutant HTT in BACHD-SF1 mice was validated with PCR analysis. Brain tissue from BACHD-SF1, BACHD, SF1 and WT mice showed the presence of the unrecombined mutant HTT allele (1050 bp) in the BACHD-SF1 and BACHD samples while the recombined mutant HTT allele (600 bp) was only detected in VMH brain samples from the BACHD-SF1 mice (Figure 1B).

**Figure 2:**
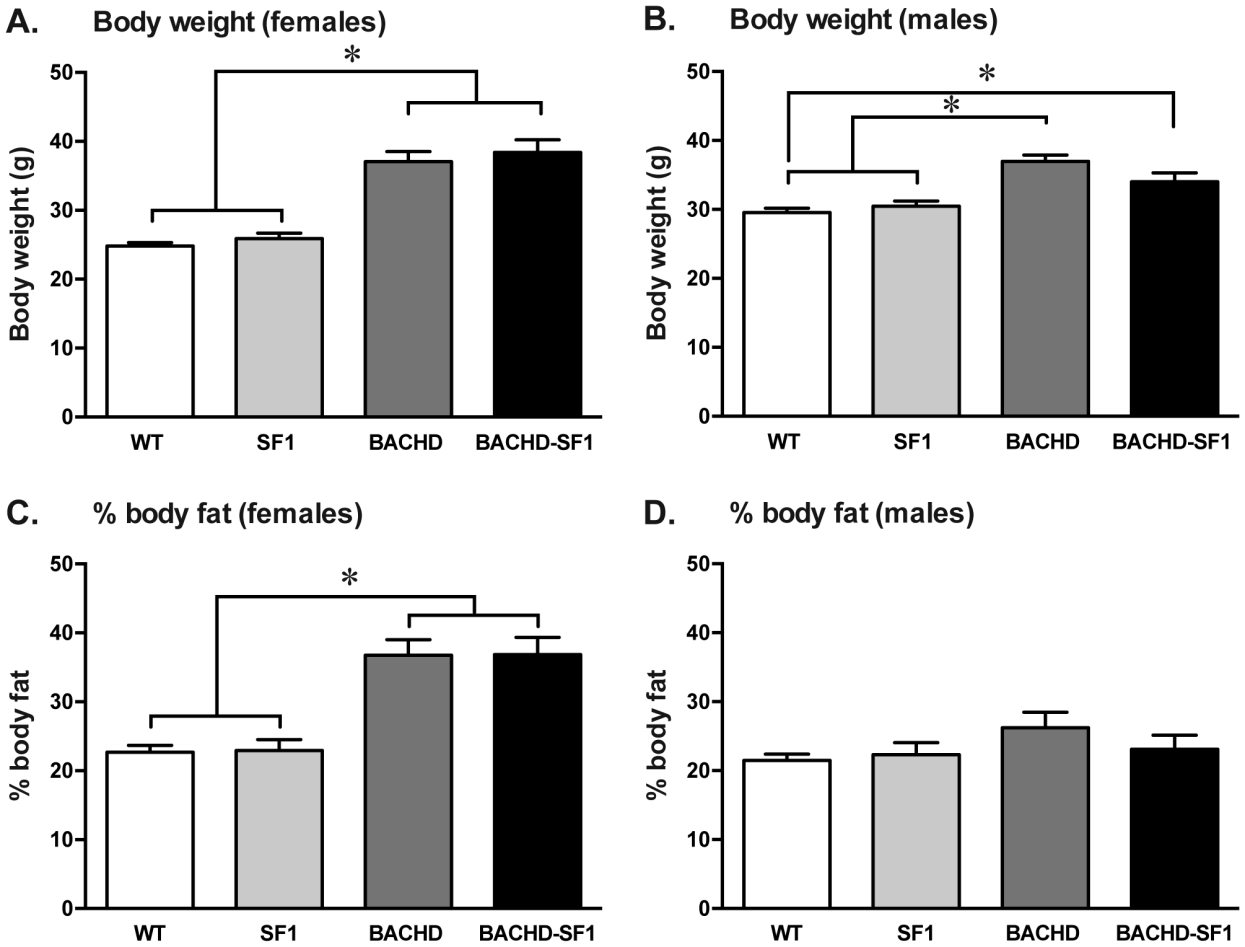
Increased body weight and body fat in 4 months old BACHD and BACHD-SF1 mice. A, B. Body weight were measured in (A) females and (B) males of each genotype. In females, the body weight of BACHD and BACHD-SF1 mice were significantly higher than WT and SF1 control mice. In males, the body weight of BACHD mice were significantly higher than WT and SF1 controls while the body weight of BACHD-SF1 mice were significantly higher than WT mice but not SF1 controls. C, D. The percentage of body fat, as determined by whole body dual energy x-ray absorptiometry (DEXA), revealed a corresponding increased body fat percentage in (C) female BACHD and BACHD-SF1 mice when compared to their WT and SF1 control counterparts but not in (D) males. Data presented as mean ± SEM (A–C, * p<0.05, one-way ANOVA with Tukey’s multiple comparisons post-hoc test, D, Kruskal-Wallis non-parametric ANOVA with Dunn’s multiple comparisons post-hoc test), n = 11–28/genotype/sex in each group. Detailed statistical analyses are outlined in Figure 2 of Statistical Results S1.

The assessment of body weight at 4 months of age showed that female BACHD-SF1 and BACHD mice were significantly different to the two female control groups and that there was no difference between BACHD-SF1 and BACHD mice (Figure 2A). Male BACHD-SF1 and BACHD mice were also significantly different to their WT littermates and there was no difference between male BACHD-SF1 and BACHD mice (Figure 2B). Similarly to previously published data using BACHD mice with FVB/N background, in the current study the body weight gain in BACHD mice was detectable already at 2 months of age in both males and females (data not shown) [14]. Hence, the inactivation of mutant HTT in SF-1 neurons in the BACHD mice does not have an effect on body weight. The percentage body fat as measured by DEXA showed a corresponding increase in body fat in both female BACHD-SF1 and BACHD mice compared to the control groups, whereas there was no difference in body fat percentage in the male groups (Figure 2C and 2D).

**Figure 3:**
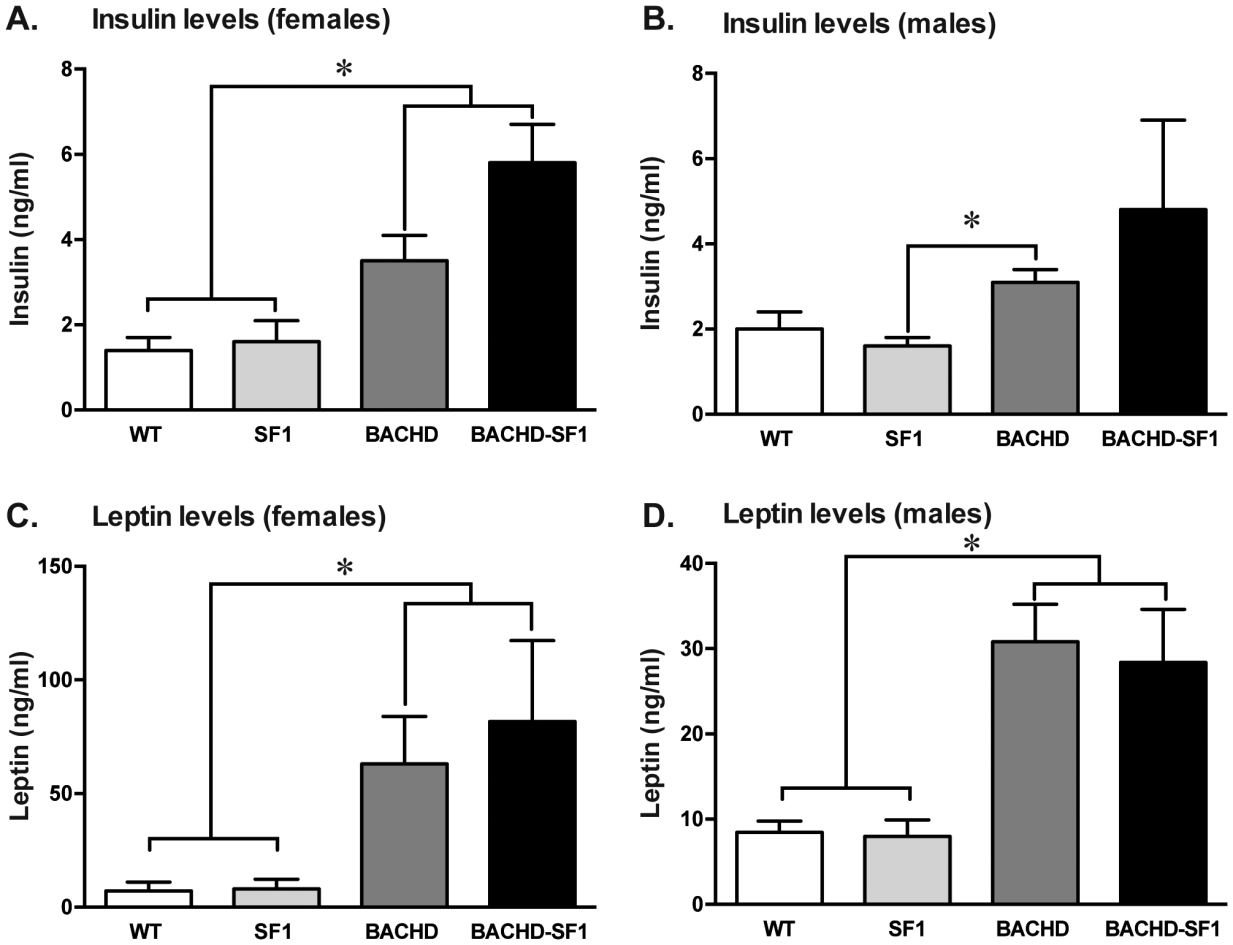
Alterations in serum insulin and leptin levels. A, B. Circulating insulin concentration was measured in blood samples from all genotypes at 4 months of age by ELISA. Serum insulin levels were significantly higher in BACHD and BACHD-SF1 females compared WT and SF1 controls while in males, only the BACHD group had significantly enhanced insulin concentration compared to SF1 controls. C, D. Serum leptin concentration as measured by ELISA was significantly higher in BACHD and BACHD-SF1 mice compared to WT and SF1 controls in both males and females. Data presented as mean ± SEM (A–B, * p<0.05, Kruskal-Wallis non-parametric ANOVA with Dunn’s multiple comparisons post-hoc test, C–D, * p<0.05, one-way ANOVA with Tukey multiple comparisons post-hoc test), n = 11–28/genotype/sex in each group. Detailed statistical analyses are outlined in Figure 3 of Statistical Results S1.

To determine if the endocrine abnormalities previously reported in the BACHD mice would be affected by inactivation of mutant HTT in SF1 neurons, serum levels of the pancreatic hormone, insulin and the adipocyte hormone, leptin were measured. In females, insulin levels in BACHD-SF1 and BACHD mice were significantly elevated compared to WT and SF1 controls (Figure 3A). In males, BACHD mice had significantly higher serum insulin levels compared to SF1 controls while insulin levels were not different between BACHD-SF1 and any other groups (Figure 3B). Leptin levels in both female and male mice were significantly higher in BACHD and BACHD-SF1 groups compared to WT and SF1 controls (Figure 3C and 3D). Taken together, the data indicate that inactivation of mutant HTT in SF1 neurons of the VMH has no effect on the metabolic phenotype as assessed by measurements of body weight, body fat and serum insulin and leptin levels in BACHD mice.

### Effects of inactivation of mutant HTT in SF1-expressing neurons on the development of anxiety-like behavior in the BACHD mice

**Figure 4:**
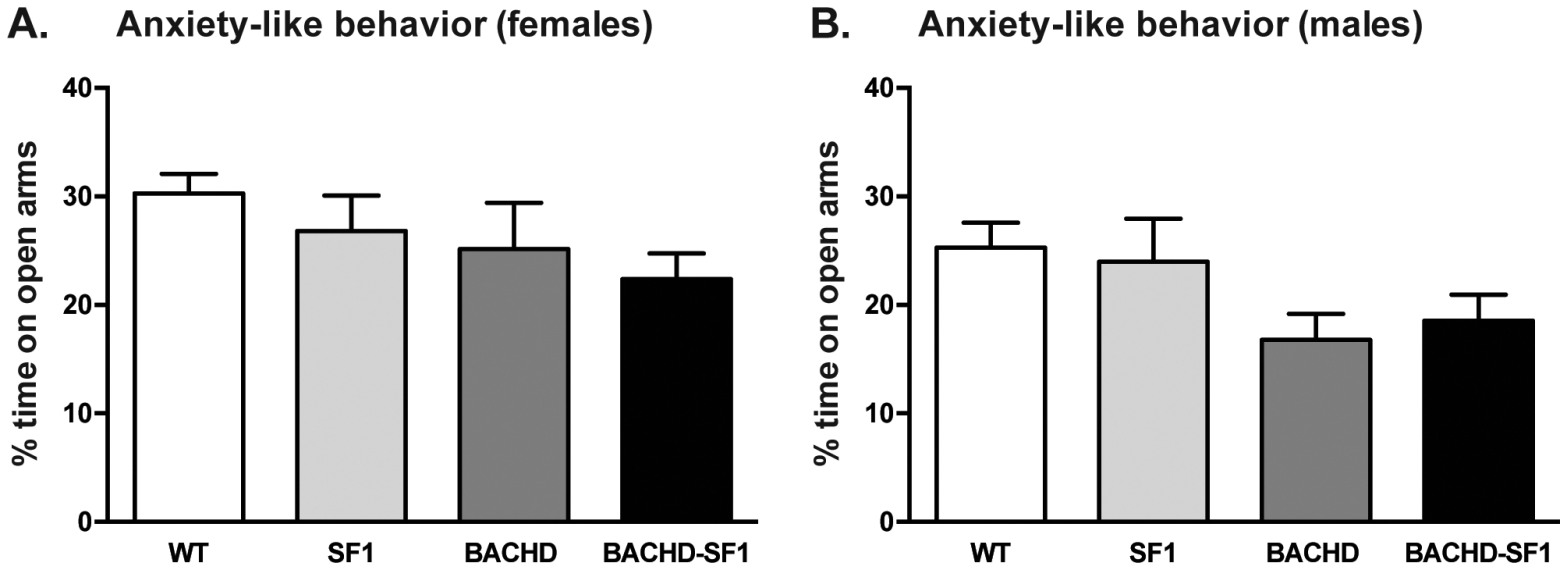
Analysis of anxiety-like behavior in BACHD-SF1 mice. Anxiety-like behavior was assessed using the elevated plus maze test and the percentage time spent on the open arms was recorded for (A) females and (B) males. There were no differences in the percentage time spent on the open arms in BACHD and BACHD-SF1 mice of both sexes compared to WT and SF1 controls. Data presented as mean ± SEM (A, Kruskal-Wallis non-parametric ANOVA with Dunn’s multiple comparisons post-hoc test, p>0.05 WT vs BACHD, WT vs BACHD-SF1, p>0.999 WT vs SF1, SF1 vs BACHD, SF1 vs BACHD-SF1, BACHD vs BACHD-SF1, B, one-way ANOVA with Tukey multiple comparisons post-hoc test), n = 11–28/genotype/sex in each group. Detailed statistical analyses are outlined in Figure 4 of Statistical Results S1.

We were then interested in examining the effect of inactivation of mutant HTT in SF1 neurons for the development of anxiety-like behavior in the BACHD mouse model. BACHD mice have previously been reported to display anxiety-like behavior already at 2 months of age as assessed using the EPM [15]. However, in this experimental setup, we found no difference in the percentage of time spent on the open arms during the 5 min test period between the different genotypes across both sexes (Figure 4A and B). As anxiety-like behavior has been found previously in 2 month old BACHD mice, a post-hoc power calculation based on the data in the present study was made in order to determine the number of additional animals that would be required to show an increased anxiety-like behavior in the BACHD mice compared to WT mice. This calculation showed that 149 BACHD and 245 WT mice would be required in order to detect a true difference with a probability of 80% (power 0.8). Hence, the lack of anxiety-like behavior in the BACHD mice in this cohort is unlikely to be due to a low number of animals in the different groups.

**Figure 5:**
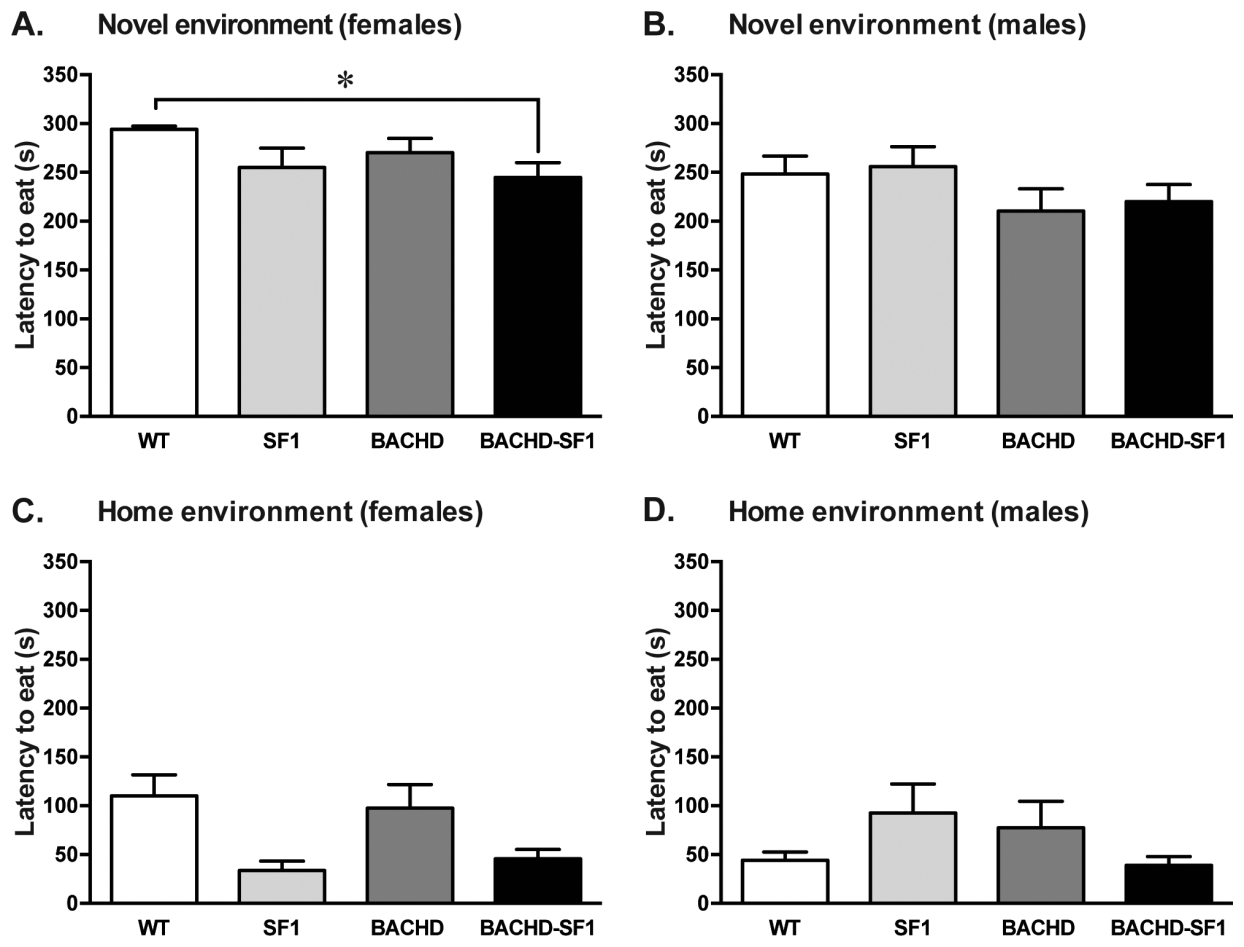
Latency to feed during the novelty-induced suppressed feeding test (NSFT). During the NSFT, the latency to feed in a novel environment as well as in the home cage was assessed. A, B. In females, BACHD-SF1 mice recorded a decreased latency to eat in the novel environment compared to WT controls but there were no differences in the male group across the genotypes. C, D. There were no differences in the latency to feed in the home environment in both sexes across the genotypes. Data presented as mean ± SEM (* p<0.05, Kruskal-Wallis non-parametric ANOVA with Dunn’s multiple comparisons test), n = 11–28/genotype/sex in each group. Detailed statistical analyses are outlined in Figure 5 of Statistical Results S1.

We also used the NSFT to assess the effect on anxiety-like behavior. We found that female BACHD-SF1 mice had a decreased latency to eat in the novel environment compared to WT mice but there were no differences between BACHD and BACHD-SF1 mice (Figure 5A). In males, there were no differences in the latency to eat in the novel environment across the different genotypes (Figure 5B). As expected, the latency to eat in the home environment was similar across the different genotypes in both females and males (Figure 5C and D). Taken together, in this cohort of mice, we found no effect of the BACHD genotype on anxiety-like behavior and a potential effect of inactivation of mutant HTT in SF1 neurons is therefore difficult to determine.

### Effects of inactivation of mutant HTT in SF1-expressing neurons on the development of depressive-like behavior in the BACHD mice

**Figure 6:**
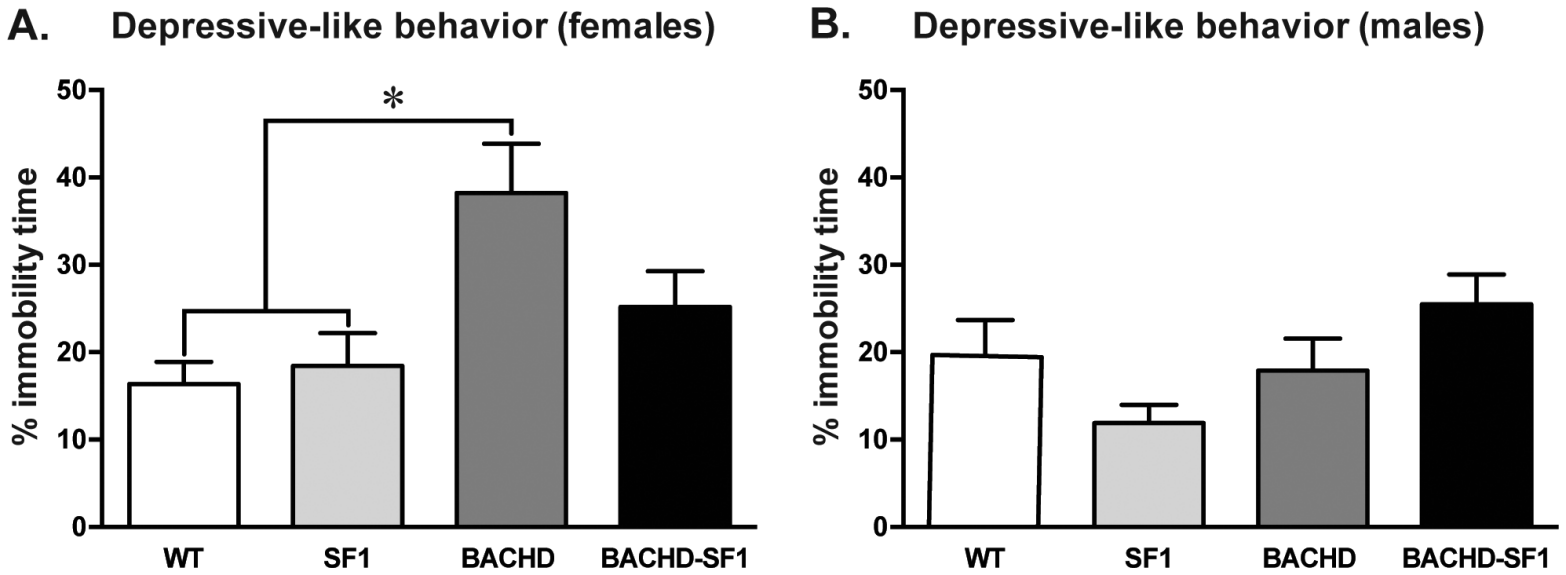
Evaluation of the depressive-like behaviors in BACHD-SF1 mice. Depressive-like behaviors were assessed using the forced swim test (FST) whereby the percentage of time spent immobile was recorded. A. In females, the percentage of time spent immobile was significantly higher in BACHD mice compared to the control groups whereas the result for BACHD-SF1 mice was not significant to any groups, suggesting a partial positive effect of inactivation of mutant HTT in SF1 neurons on this behavior. B. There were no differences in the percentage of time spent immobile across all genotypes in males. Data presented as mean ± SEM (* p<0.05, one-way ANOVA with Tukey multiple comparisons post-hoc test), n = 11–28/genotype/sex in each group. Detailed statistical analyses are outlined in Figure 6 of Statistical Results S1.

To assess the effect on depressive-like behavior in this study, mice were subjected to the FST. Consistent with previous reports of increased immobility in BACHD mice as an indication of depressive-like behavior [15], [32], [36], BACHD females exhibited a significant increase in the percentage of time spent immobile compared to WT and SF1 control littermates (Figure 6A; one-way ANOVA followed by post-hoc Tukey, p = 0.0004 BACHD vs WT, p = 0.014 BACHD vs SF1). There were no significant differences between BACHD-SF1 and BACHD mice, indicating that SF1 neurons alone are not sufficient for contributing to the depressive-like phenotype (one-way ANOVA, post-hoc Tukey, p = 0.11 BACHD vs BACHD-SF1). However, there were also no significant differences between BACHD-SF1 and the control groups. We therefore performed a power calculation to determine the additional number of animals required to prove a potential rescue of the depressive-like behavior and found that 27 BACHD and 27 BACHD-SF1 mice would be required to find a true difference between the two BACHD groups with a probability of 80% (power 0.8). In the present design, 17 female BACHD and 17 BACHD-SF1 mice were included. No differences were detected in the percentage of time spent immobile between the genotypes in males (Figure 6B). Our data suggests that inactivation of mutant HTT in SF1 neurons may have a partial effect on the development of depressive-like behavior in female BACHD mice.

### No change in motor activity in mice after conditional deletion of mutant HTT from SF1-expressing neurons in BACHD mice

**Figure 7:**
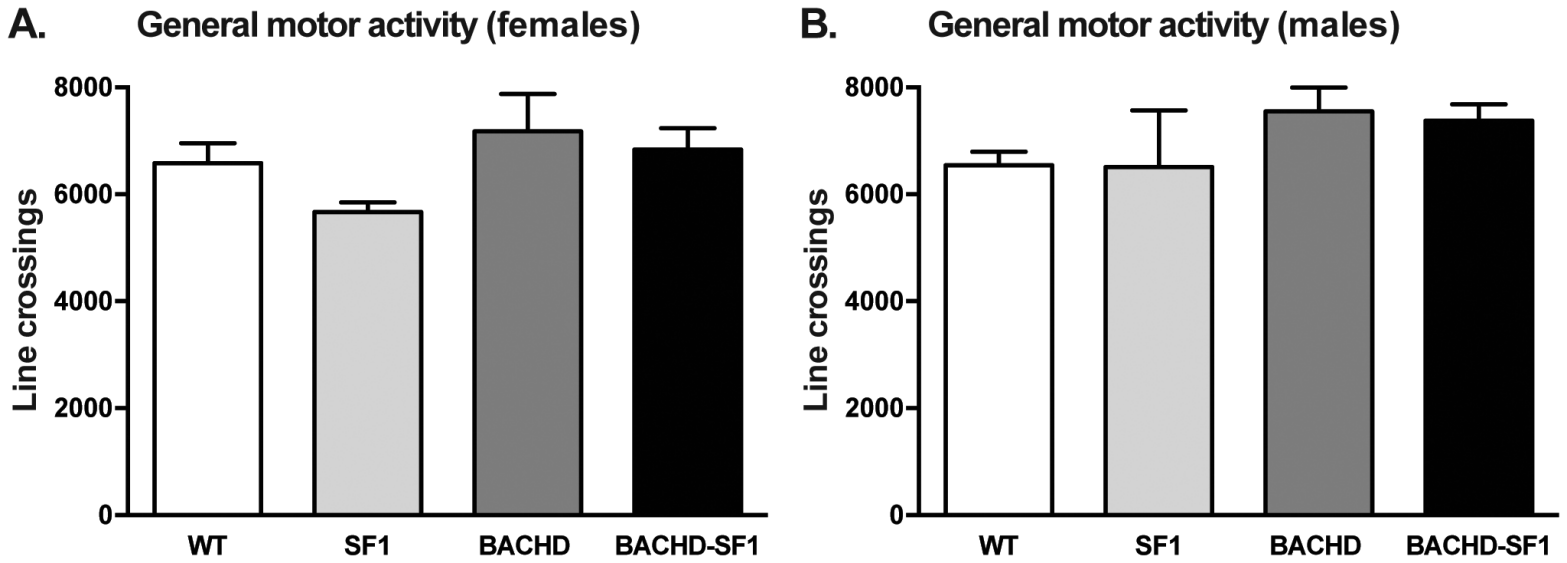
Assessment of motor activity in BACHD-SF1 mice. Motor activity was assessed using the open field test at 4 months of age. Mice were placed in the open field chamber for 1 hour and the total number of line crossings and line crossings in the center were recorded. There were no differences in the number of line crossings (A, B) and in the number of line crossings in the center (C, D) made by BACHD and BACHD-SF1 mice of both sexes compared to their respective WT comparisons. Data presented as mean ± SEM (Kruskal-Wallis non-parametric ANOVA with Dunn’s multiple comparisons test), n = 11–28/genotype/sex in each group. Detailed statistical analyses are outlined in Figure 7 of Statistical Results S1.

General locomotor activity was measured with the OF test. No significant differences were detected in the number of line crossings between BACHD-SF1, BACHD, SF1 and WT controls in both sexes, indicating an absence of motor dysfunction at this age in all groups (Figure 7A and B).

## Discussion

Non-motor symptoms and signs are common in neurodegenerative HD. As they sometimes manifest before the motor dysfunction, their underlying biological mechanisms may be part of early steps in the pathogenesis of the disease. Identification of the neural substrates responsible for these aspects of the disease may therefore be important in the search for novel targets in the development of potential disease modifying treatments. The hypothalamus is an area of the brain involved in the regulation of emotion, metabolism and sleep, functions that are part of non-motor spectra of HD [4]. Imaging studies based on PET and MRI have indeed implicated that the hypothalamus is affected early on in HD gene carriers [12], [13]. Recent studies using experimental models of HD have established a link between expression of mutant HTT in the hypothalamus and the development of metabolic dysfunction as well as depressive-like behavior [14], [15]. In the present study, we were interested in further dissecting out which specific hypothalamic nuclei/circuitry would be responsible for this effect. We therefore inactivated mutant HTT in SF1-expressing neurons in the VMH, which has been implicated in the regulation of metabolism and emotion [30], [37], [38]. However, deletion of mutant HTT from these neurons only had a partial positive effect on the depressive-like behavior in female BACHD mice and did not influence other behavioral or metabolic parameters. Hence, dysfunction of neural circuitries involving the VMH is unlikely to be important for the development of the non-motor phenotype in BACHD mice.

Although several models have been developed to study psychiatric disturbances in clinical HD, modelling the full spectrum of symptoms remains a challenge. Several HD mouse models have shown psychiatric abnormalities with the different behavioral tests, thus recapitulating some of the aspects of clinical HD [39], [40]. The BACHD model has previously been shown to display depressive-like behavior in the FST as early as 2 months of age and independent of sex [15]. In this cohort of mice, we replicated this finding only in female BACHD mice. Clear sex differences in the psychiatric-like behavior in BACHD mice have not been reported previously but in other HD mouse models such as the R6/1 model, depressive-like behavior has been reported only in female mice [34], [41], [42]. One possible explanation for this discrepancy and a potential caveat of the study is that the mice used were on a mixed FVB/N and C57BL6/J background and it is well known that strain differences can have major effects on the phenotype in mice [43]–[45]. It is therefore possible that the depressive-like phenotype in BACHD mice is stronger in female compared to male mice, as it appears more resistant to this potential strain effect. Interestingly, although the FST result in female BACHD-SF1 mice was not different to BACHD mice, it was also no longer different to the result in the control groups, suggesting a partial positive effect of conditional deletion of mutant HTT from SF1-expressing neurons. We therefore performed a power calculation to determine how many additional mice in each group would have been necessary to see a significant difference between BACHD and BACHD-SF1 mice. We find that an additional 10 animals in the BACHD and BACHD-SF1 groups respectively would be required to prove that mutant HTT expression in SF1 neurons is necessary for the depressive-like phenotype with a probability of 80%. Although SF1 neurons and the wider VMH have been implicated in anxiety-like but not depressive-like behaviors [46], this finding suggests that the expression of mutant HTT in SF1 neurons may play a role for the development of the depressive-like phenotype. This is not surprising since the neurons of the VMH project to brain regions and other parts of the limbic system involved with anxiety and affective behaviors [47]. In addition, a recent clinical study from patients with SF1 (Nr5a1) gene mutations show psychiatric symptoms including excessive anxiety and/or depression [48], suggesting that SF1 and the VMH may have roles in both anxiety- and depressive-like behaviors. Nevertheless, we acknowledge that the power calculation is a mathematical estimation and further studies are warranted to determine if mutant HTT expression in SF1 neurons is important for the depressive-like behavior.

SF1-neurons in the VMH have been shown to regulate anxiety-like behaviors as well as genes mediating anxiety [23], [46]. Studies with central nervous system-specific SF1 knockout mice displayed an altered expression of genes implicated in anxiety-behavior in the VMH including BDNF, Crhr2, and Ucn 3 [23]. We have demonstrated previously that the BACHD mouse model of HD on the FVB/N strain exhibit anxiety-like behavior already at 2 months of age using the EPM [15]. However, in the present study, we found no evidence for an anxiety-like phenotype in BACHD or BACHD-SF1 mice as assessed using both the EPM and NSFT. Power analysis of the results from the EPM revealed that a very large sample size would be required to substantiate an anxiety-like phenotype in BACHD mice in this cohort, mostly due to a large individual variation within the group. One possible explanation for this discrepancy is again that the mice used were on mixed FVB/N and C57BL6/J background. Several studies including our own have reported anxiety-like behaviors on transgenic models expressing the full-length mutant HTT on the FBV/N strain [15], [36]. Phenotypic abnormalities have been associated with strain differences of the YAC128 mice [43], but precisely how these strain differences on a mixed background affect the development of the anxiety-like behaviors in BACHD mice remains unknown.

The VMH has long been regarded to be one of the critical hypothalamic nuclei responsible for the control of energy homeostasis [20], [27], [37]. Early lesion studies into the VMH described hyperphagic obesity and revealed the importance of this hypothalamic nucleus in regulating food and energy balance [49]–[51]. There is compelling evidence from studies using various full-length mutant HTT transgenic models such as BACHD and YAC128 demonstrating increased body weight and body fat which is thought to correspond to the increased leptin levels [16], [31], [52]. Several mechanisms underlying this phenotype have been proposed. Overexpression of the HTT protein independent of the mutation has been suggested as one possibility [52]. However, our previous work have demonstrated that inactivation of mutant HTT in hypothalamic neurons alone prevented the development of the metabolic phenotype of BACHD mice [14]. In undertaking the present study, we sought to determine if the conditional deletion of mutant HTT in SF1-expressing neurons during embryonic development might rescue the metabolic phenotype. However, we found no evidence that mutant HTT expression in SF1 neurons is critical for regulating the increased body weight phenotype or elevated leptin and insulin levels, suggesting that the metabolic phenotype is not attributed to mutant HTT expression in SF1 neurons. Concomitantly, we found that the increased body weight corresponded to a similar increase in body fat assessed in females but not in males. The reason for this discrepancy is unclear since leptin levels remain elevated in BACHD and BACHD-SF1 males. A possible explanation could be the high variability within the group associated with the animals having ad libitum access to food and water. This issue with high group variation can be rectified in future studies by fasting animals prior to blood collection for insulin and leptin measurements.

In conclusion, we find little support for the notion that mutant HTT expression in VMH SF1 neurons is responsible for the development of the psychiatric and metabolic impairments in BACHD mice. However, the deletion of mutant HTT from SF1-expressing neurons occurred from birth and the lack of an effect on the behavioral phenotype may reflect compensatory mechanisms proceeding from the time of mutant HTT deletion during embryonic development. It is possible that the adaptive response in the brain that occurs during embryonic development could lead to the formation of a compensatory circuitry. The degree to which this happens remains unclear. Also, taking into account that the VMH is a heterogeneous nucleus made up of anatomically distinct sub-nuclei and the fact that SF1 itself is not expressed throughout the entire VMH [24], [25], the metabolic and psychiatric impairments could however be mediated by non-SF1 neurons also located in the VMH. Further studies using viral vector injections carrying Cre into the VMH in adult mice and/or using SF1 mini promoters may be necessary to determine if the VMH and SF1 neurons are critical for the development of the psychiatric and metabolic impairments in HD without the potential confound from compensatory mechanisms. Overall, these aspects emphasize the complexity of the hypothalamic neuronal system and suggest that it is perhaps too simplistic to consider whole populations of hypothalamic neurons separately in investigations aiming at determining the underlying neural mechanisms of the non-motor aspects of HD.

## Supporting Information

Figure S1 **Generation of BACHD-SF1 mouse line.** Male BACHD mice, expressing human full-length HTT with 97 polyQ and floxed exon1 (Ex1-97Q) were crossed with female mice expressing Cre under the SF1 promoter (SF1). As a result of the crossing four genotypes were obtained: WT animals expressing mouse endogenous WT HTT, SF1 animals expressing Cre, BACHD mice expressing human full-length mutant HTT with 97 polyQ and BACHD-SF1 animals expressing Cre and mutant HTT. In the BACHD-SF1 animals, the presence of Cre induces the recombination of the floxed gene resulting in a conditional deletion of mutant HTT in the SF1 neurons.
(TIF)

Statistical Results S1 (DOCX)
