# Supplementary figures and images for: Effects of Deletion of Mutant Huntingtin in Steroidogenic Factor 1 Neurons on the Psychiatric and Metabolic Phenotype in the BACHD Mouse Model of Huntington Disease
Source: PLoS One. 2014 Oct 1;9(10):e107691. doi: 10.1371/journal.pone.0107691 (PMC4182678; doi:10.1371/journal.pone.0107691)

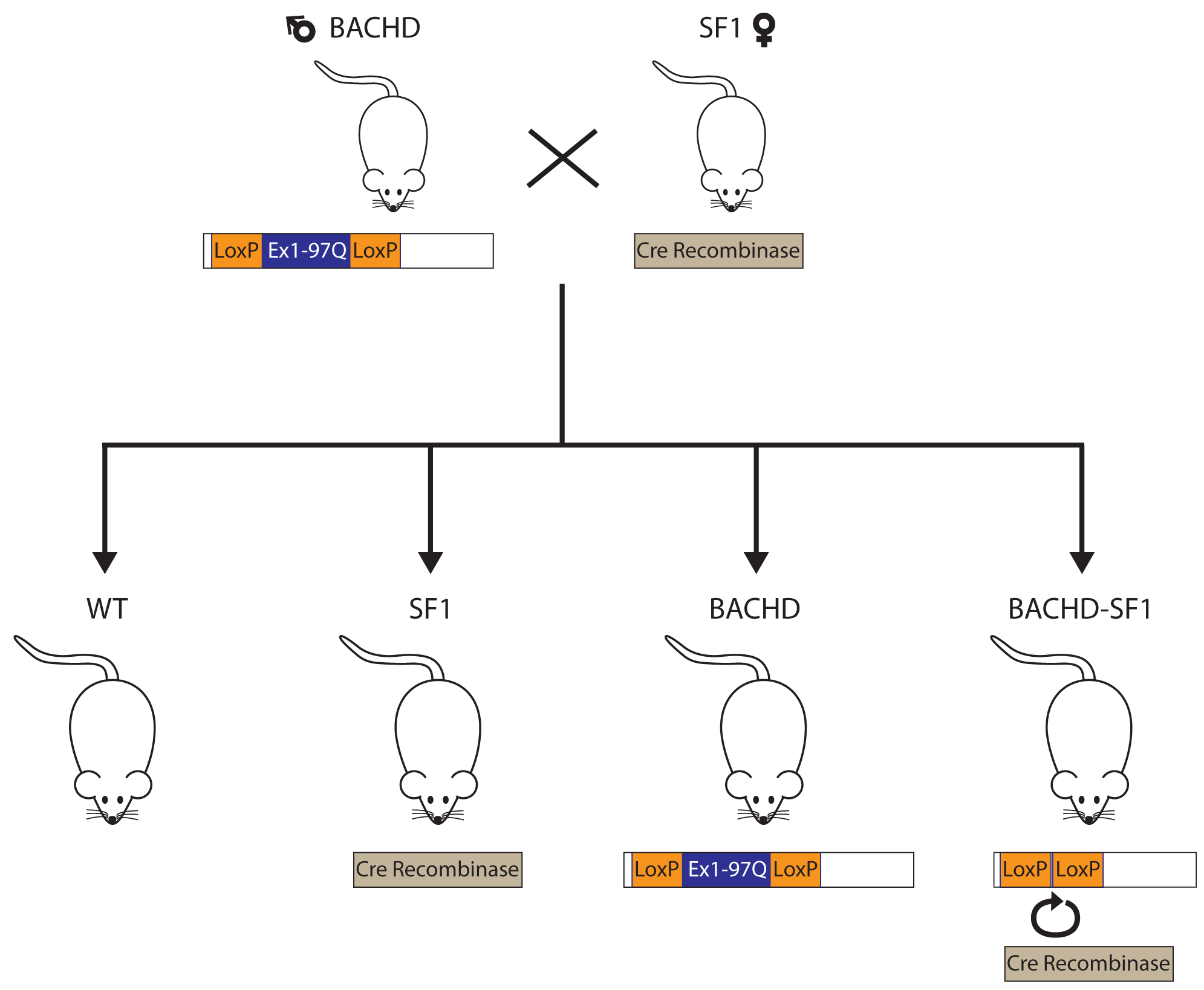

Supplement: Figure S1 — Generation of BACHD-SF1 mouse line. Male BACHD mice, expressing human full-length HTT with 97 polyQ and floxed exon1 (Ex1-97Q) were crossed with female mice expressing Cre under the SF1 promoter (SF1). As a result of the crossing four genotypes were obtained: WT animals expressing mouse endogenous WT HTT, SF1 animals expressing Cre, BACHD mice expressing human full-length mutant HTT with 97 polyQ and BACHD-SF1 animals expressing Cre and mutant HTT. In the BACHD-SF1 animals, the presence of Cre induces the recombination of the floxed gene resulting in a conditional deletion of mutant HTT in the SF1 neurons. (TIF) [file pone.0107691.s001.tif]
