# Supplementary material for: Effects of Deletion of Mutant Huntingtin in Steroidogenic Factor 1 Neurons on the Psychiatric and Metabolic Phenotype in the BACHD Mouse Model of Huntington Disease
Source: PLoS One. 2014 Oct 1;9(10):e107691. doi: 10.1371/journal.pone.0107691 (PMC4182678; doi:10.1371/journal.pone.0107691)
Supplement: Statistical Results S1 — (DOCX) [file pone.0107691.s002.docx]

**Statistical results S1**

In the Kolmogorov–Smirnov test, p-value >0.05 indicates normal distribution of the data.

In 1-factor ANOVA and Kruskal–Wallis test statistical significance is considered when p-values <0.05. For *post hoc* tests only p-values <0.05 are reported.

**Figure 2:**

A. Body weight (females)

Kolmogorov–Smirnov test:

- WT: p>0.1000
- SF1: p>0.1000
- BACHD: p>0.1000
- BACHD-SF1: p>0.1000

1-factor ANOVA at 4 months:

F_(3,69)_= 38.89, p<0.0001

Tukey post-hoc test:

WT vs BACHD p< 0.0001

WT vs BACHD-SF1 p< 0.0001

SF1 vs BACHD p< 0.0001

SF1 vs BACHD-SF1 p< 0.0001

B. Body weight (males)

Kolmogorov–Smirnov test for normal distribution:

- WT: p>0.1000
- SF1: p=0.0725
- BACHD: p>0.1000
- BACHD-SF1: p>0.1000

1-factor ANOVA at 4 months:

F_(3,57)_= 11.66, p<0.0001

Tukey post-hoc test:

WT vs BACHD p< 0.0001

WT vs BACHD-SF1 p=0.0057

SF1 vs BACHD p< 0.0005

C. % body fat (females)

Kolmogorov–Smirnov test:

- WT: p>0.1000
- SF1: p>0.1000
- BACHD: p>0.1000
- BACHD-SF1: p>0.1000

1-factor ANOVA at 4 months:

F_(3,69)_ = 20.22, p<0.0001

Tukey post-hoc test:

WT vs BACHD p< 0.0001

WT vs BACHD-SF1 p< 0.0001

SF1 vs BACHD p< 0.0001

SF1 vs BACHD-SF1 p< 0.0001

D. % body fat (males)

Kolmogorov–Smirnov test:

- WT: p>0.1000
- SF1: p>0.1000
- BACHD: p=0.0362
- BACHD-SF1: p>0.1000

Kruskal–Wallis 1-factor ANOVA at 4 months:

H=2.031, p=0.5661

**Figure 3:**

A. Insulin (females)

Kolmogorov–Smirnov test:

- WT: p>0.1000
- SF1: p=0.0009
- BACHD: p=0.0470
- BACHD-SF1: p>0.1000

Kruskal–Wallis 1-factor ANOVA at 4 months:

H=22.99, p<0.0001

Dunn’s post-hoc test:

WT vs BACHD p=0.0314

WT vs BACHD-SF1 p=0.0005

SF1 vs BACHD p=0.0498

SF1 vs BACHD-SF1 p=0.0009

B. Insulin (males)

Kolmogorov–Smirnov test:

- WT: p>0.1000
- SF1: p>0.1000
- BACHD: p=0.0787
- BACHD-SF1: p=0.0013

Kruskal–Wallis 1-factor ANOVA at 4 months:

H=9.173, p=0.0271

Dunn’s post-hoc test:

SF1 vs BACHD p=0.0270

C. Leptin (females)

Kolmogorov–Smirnov test:

- WT: p>0.1000
- SF1: p>0.1000
- BACHD: p>0.1000
- BACHD-SF1: p>0.1000

1-factor ANOVA at 4 months:

F_(3,36)_ = 33.61, p<0.0001

Tukey post-hoc test:

WT vs BACHD p< 0.0001

WT vs BACHD-SF1 p< 0.0001

SF1 vs BACHD p< 0.0001

SF1 vs BACHD-SF1 p< 0.0001

D. Leptin (males)

Kolmogorov–Smirnov test:

- WT: p=0.0854
- SF1: p>0.1000
- BACHD: p>0.1000
- BACHD-SF1: p>0.1000

1-factor ANOVA at 4 months:

F_(3,36)_ = 6.667, p=0.0011

Tukey post-hoc test:

WT vs BACHD p=0.0113

WT vs BACHD-SF1 p=0.0282

SF1 vs BACHD p=0.0095

SF1 vs BACHD-SF1 p=0.0239

**Figure 4:**

A. Anxiety-like behavior (females)

Kolmogorov–Smirnov test:

- WT: p>0.1000
- SF1: p>0.1000
- BACHD: p=0.0002
- BACHD-SF1: p>0.1000

Kruskal–Wallis 1-factor ANOVA at 4 months:

H=10.14, p=0.0174

B. Anxiety-like behavior (males)

Kolmogorov–Smirnov test:

- WT: p>0.1000
- SF1: p>0.1000
- BACHD: p>0.1000
- BACHD-SF1: p>0.1000

1-factor ANOVA at 4 months:

F_(3,59)_ = 2.340, p=0.0825

**Figure 5:**

A. Novel environment (females)

Kolmogorov–Smirnov test:

- WT: p<0.1000
- SF1: p=0.0074
- BACHD: p<0.1000
- BACHD-SF1: p<0.1000

Kruskal–Wallis 1-factor ANOVA at 4 months:

H=9.087, p=0.0282

Dunn’s post-hoc test:

WT vs BACHD-SF1 p=0.0375

B. Novel environment (males)

Kolmogorov–Smirnov test:

- WT: p=0.0024
- SF1: p=0.0015
- BACHD: p>0.1000
- BACHD-SF1: p=0.0896

Kruskal–Wallis 1-factor ANOVA at 4 months:

H=4.166, p=0.2441

C. Home environment (females)

Kolmogorov–Smirnov test:

- WT: p<0.1000
- SF1: p=0.0117
- BACHD: p=0.0013
- BACHD-SF1: p>0.1000

Kruskal–Wallis 1-factor ANOVA at 4 months:

H=5.923, p=0.1154

D. Home environment (males)

Kolmogorov–Smirnov test:

- WT: p>0.1000
- SF1: p=0.0104
- BACHD: p=0.0089
- BACHD-SF1: p=0.0002

Kruskal–Wallis 1-factor ANOVA at 4 months:

H=2.250, p=0.5221

**Figure 6:**

A. Depressive-like behavior (females)

Kolmogorov–Smirnov test:

- WT: p>0.1000
- SF1: p>0.1000
- BACHD: p=0.0534
- BACHD-SF1: p>0.1000

1-factor ANOVA at 4 months:

F_(3,70)_ = 6.467, p=0.0514

Tukey post-hoc test:

WT vs BACHD p=0.0004

SF1 vs BACHD p=0.0144

B. Depressive-like behavior (males)

Kolmogorov–Smirnov test:

- WT: p>0.1000
- SF1: p>0.1000
- BACHD: p>0.1000
- BACHD-SF1: p>0.1000

1-factor ANOVA at 4 months:

F_(3,58)_ = 2.261, p=0.0909

**Figure 7:**

A. General motor activity (females)

Kolmogorov–Smirnov test:

- WT: p=0.0003
- SF1: p>0.1000
- BACHD: p=0.0001
- BACHD-SF1: p>0.1000

Kruskal–Wallis 1-factor ANOVA at 4 months:

H=5.637, p=0.1307

B. General motor activity (males)

Kolmogorov–Smirnov test:

- WT: p>0.1000
- SF1: p=0.0005
- BACHD: p>0.1000
- BACHD-SF1: p>0.1000

Kruskal–Wallis 1-factor ANOVA at 4 months:

H=9.302, p=0.0255
